# Case Report: Intrahepatic arterioportal fistula (IAPF)—a rare cause of hemobilia

**DOI:** 10.3389/fmed.2026.1777550

**Published:** 2026-02-23

**Authors:** Ye Wang, Wangde Jin, Xu Jiang, Xinglin Jin

**Affiliations:** Department of Hepatobiliary Surgery, Yanbian University Hospital, Yanji, China

**Keywords:** case report, ERCP, hemobilia, intrahepatic arterioportal fistula, transcatheterarterial embolization

## Abstract

Intrahepatic arterioportal fistula is a rare vascular malformation, and cases of hemobilia due to it are extremely rare. This article reports a rare case of hemobilia due to intrahepatic arterioportal fistula in an elderly woman. Upon admission, the patient presented with sporadic upper abdominal pain lasting 3 days. The initial diagnosis of choledocholithiasis was made following a review of the upper abdominal Computed Tomography (CT) scan from a previous hospital and our hospital's Magnetic Resonance Cholangiopancreatography (MRCP). Hemobilia and sedimentary clot were identified during ERCP microscopy. Post-ERCP imaging, specifically CT enhancement, revealed an intrahepatic arterioportal fistula. This finding, in conjunction with insights from a multidisciplinary consultation, led to treatment via transcatheter arterial embolization (TAE). The patient recovered well postoperatively with cessation of bleeding. This case demonstrates IAPF as a rare cause of biliary bleeding and the diagnostic value of imaging for IAPF, which provides reference for the diagnosis and treatment of complex biliary diseases in elderly patients.

## Introduction

Hemobilia (HB) is a rare cause of upper gastrointestinal bleeding due to medical injury, trauma, malignancy, or vascular malformations, and is diagnosed with increasing frequency due to advances in endoscopic procedures (1). Intrahepatic arterioportal fistula is a rare vascular malformation that can be congenital (e.g., developmental anomalies) or acquired (e.g., trauma, tumors, and medical-origin injuries), and is mainly characterized by portal hypertension, hemobilia, and hepatic function abnormalities (2). Its diagnosis relies on imaging [CT/Magnetic Resonance Imagin (MRI) enhancement, angiography], and treatment options include surgery (fistula repair, hepatic lobectomy) and interventional embolization (TAE). For elderly patients, interventional therapy has become the preferred option due to its minimally invasive nature and low impact on liver function.

## Case description

**Figure 1:**
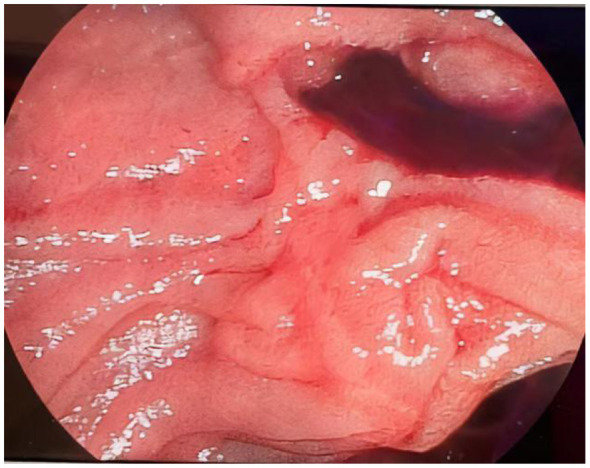
A strip-shaped blood clot from the biliary tract was found at the opening of the major duodenal papilla of the duodenum under duodenoscopy.

**Figure 2:**
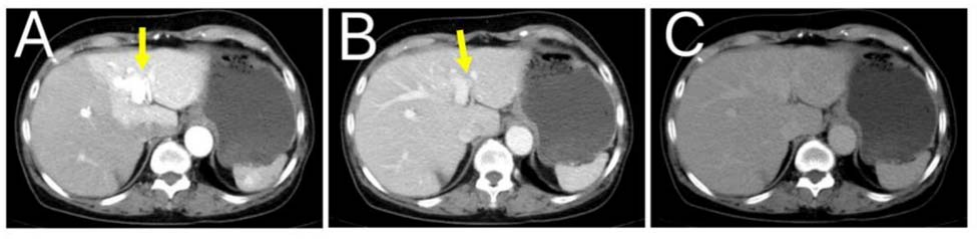
**(A)** Denotes the arterial phase, **(B)** indicates the portal venous phase, and **(C)** signifies the equilibrium phase. The yellow arrow highlights the intrahepatic arterioportal fistula. Enhanced scanning shows obvious enhancement foci in the arterial phase, slightly high density in the portal phase and equilibrium phase, which is basically synchronous with the enhancement of adjacent portal veins. The left branch of the portal vein is widened, and the portal vein is early visible. Patchy abnormal perfusion in the arterial phase can be seen in the surrounding liver parenchyma.

**Figure 3:**
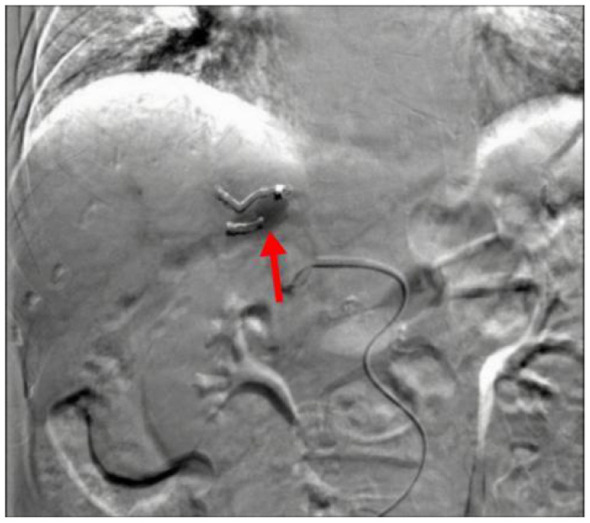
As indicated by the red arrow in the figure, transcatheter hepatic artery embolization was performed using coils and gelatin sponge particles.

**Figure 4:**
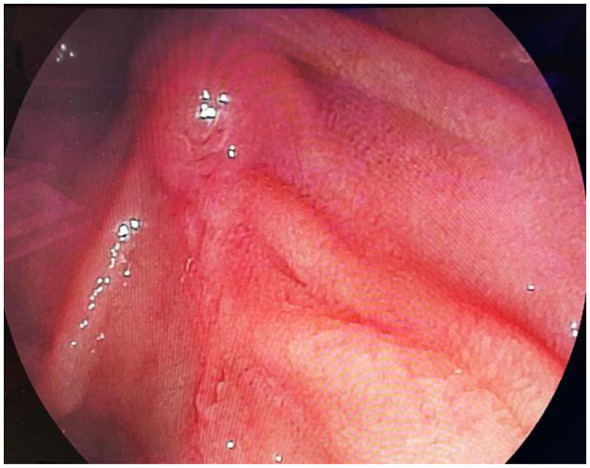
One week after the operation, duodenoscopy reexamination showed no signs of continued hemobilia.

A 73-year-old female patient was admitted to the hospital with intermittent upper abdominal pain persisting for 3 days. The patient has a 10-year history of cerebral infarction, hypertension, and heart disease. She denies any history of liver diseases, including hepatitis and cirrhosis, as well as any history of abdominal trauma, previous abdominal procedures (such as puncture or traditional Chinese acupuncture), or surgeries. There is no relevant family or genetic history. The patient mentioned experiencing black stools a few days prior, albeit in small quantities, and this issue has not recurred. Upon physical examination, tenderness was noted in the right upper abdomen, but there was no rebound tenderness, muscle rigidity, hepatosplenomegaly, varicose veins, spider nevi, or yellowish discoloration of the skin, mucous membranes, or sclera. Laboratory tests: red blood cell counts of 3.75 × 1012/L (3.50–5.50), hemoglobin levels of 113g/L (110–160), and hematocrit at 34% (36–50), alanine aminotransferase (ALT)206U/L (0–40), aspartate aminotransferase (AST) 99U/L (0–40), alkaline phosphatase (ALP) 212U/L (42–140), gamma-glutamyltransferase (GGT) 600U/L (8–58), serum total bilirubin 22.6 μmol/L (5.1–25.6) and serum direct bilirubin 15.7 μmol/L (1.7–6.8). The patient did not have a bowel movement within 2 days of hospitalization, which precluded routine stool sampling. A CT scan of the upper abdomen from a previous hospital revealed the presence of sand-like stones in the bile duct, along with dilation of the common bile duct. The completion of MRCP also indicates multiple sand-like stones in the common bile duct accompanied by mild dilation of the intrahepatic and extrahepatic bile ducts. ERCP was performed on the patient to facilitate stone removal. Duodenoscopy revealed no abnormalities in the esophagus. Upon entering the stomach, old bloodstains were observed on the mucosa; however, no active bleeding points, ulcers, or erosions were present. A strip-shaped blood clot was found at the opening of the major duodenal papilla in the descending segment of the duodenum (Figure 1). Following successful intubation, a diffuse irregular filling defect was noted in the common bile duct. This defect exhibited an irregular shape, rough edges, and flocculent changes, with delayed contrast agent emptying. As the columnar air sac gradually expanded, a strip-shaped blood clot continued to flow from the duodenal papilla. The family opted against the use of the SpyGlass biliary subscope for personal reasons, which precluded further exploration of the biliary tract. During the procedure, it was determined that the remaining blood clot might provide a protective effect; thus, the decision was made to refrain from complete removal due to the potential risk of rebleeding. The operation concluded with the insertion of a nasobiliary duct to alleviate pressure within the bile duct and ensure adequate drainage. However, the patient independently removed the nasobiliary duct 6 h post-operation. To investigate the cause of HB after the operation, an contrast enhanced CT (CECT) (Figure 2) scan of the upper abdomen was performed, which indicated abnormal enhancement foci in the square lobe of the liver. Vascular malformations (Intrahepatic arterioportal fistula) were considered. At this point, the blood routine test indicates: red blood cell counts of 3.03 × 1012/L, hemoglobin levels of 92 g/L, and hematocrit at 27.5%. After comprehensively considering the patient's auxiliary examinations and medical history and ruling out any secondary causes of IAPF, Congenital Intrahepatic arterioportal fistula (CIAPF) was diagnosed with the aid of enhanced CT. CIAPF arises from the abnormal development of the hepatic vascular system during embryogenesis, leading to atypical connections between the hepatic artery and the portal vein. After undergoing MDT for further treatment, interventional embolization therapy was chosen. A puncture needle was inserted into the right femoral artery, and a vascular sheath was placed. A 5F hepatography catheter was used to insert the superior mesenteric artery and the proper hepatic artery through the abdominal trunk at the lower edge of the 12th thoracic artery for angiography. An arteriovenous fistula was observed in the left lobe of the liver, suggesting bleeding caused by the arteriovenous fistula. Due to vascular malformations, a microcatheter was inserted into the artery of the arteriovenous fistula under the guidance of a microguide wire. Embolization was performed with six coils, and the embolization was further consolidated with gelatin sponge particles (700–1,000 μm) (Figure 3). The tube was removed, and hemostasis was achieved by compressing the wound for 10 min before applying pressure dressings. The operation went smoothly. During the operation, the patient's vital signs remained stable and there was no obvious discomfort. Postoperative dynamic blood routine monitoring was conducted, and the blood routine on the same night indicated: red blood cell counts of 2.94 × 1012/L, hemoglobin levels of 87 g/L, and hematocrit at 27.4%. The blood routine test the next day indicated: red blood cell counts of 2.85 × 1012/L, hemoglobin levels of 88 g/L, and hematocrit at 26.8%. One week after the operation, a duodenoscopy follow-up was conducted, and no further hemobilia was observed (Figure 4).

## Discussion

IAPF is a functional or organic connection between the portal venous system and the branches of the hepatic artery (3), and it is a rare cause of HB. IAPF may be caused by trauma, malignant tumors or iatrogenic injury, and in some cases, it is hereditary (4). To our knowledge, this is an extremely rare report in English literature about HB caused by IAPF. IAPF was first reported approximately 50 years ago and is now defined as the intrahepatic traffic between the hepatic artery and the portal vein system (5). HB is often not given priority before endoscopic examination and may be poorly managed over the long term. Therefore, doctors must be aware of the various causes and manifestations of HB, including less common ones. Once HB is detected through gastroduodenoscopy, contrast enhanced CT and selective hepatic artery angiography remain accurate diagnostic tools for identifying the source of bleeding. It usually helps define and locate arterial lesions, such as IAPF (6). The contrast-enhanced CT results of IAPF typically include: significant enhancement in the arterial phase, slightly high density in the portal phase and equilibrium phase, which is basically synchronous with the enhancement of adjacent portal veins. The portal vein may be widened and early imaging is possible. IAPF treatment includes: percutaneous transarterial embolization, surgical ligation of affected hepatic arteries, partial hepatectomy, etc. Interventional radiotherapy is regarded as the first choice (6, 7). Multidisciplinary collaboration (interventional department, radiology department, general surgery department) can optimize diagnosis and treatment decisions and enhance treatment safety. Given the patient's prior history of melena, we propose that the HB may stem from the rupture of the vascular plexus surrounding the biliary tract, which is likely due to elevated pressure at the porta hepatis. This rupture would allow blood to enter the bile duct, with a portion subsequently flowing into the duodenum and entering the digestive tract. In addition, the vascular walls near the fistula are prone to erosion or pseudoaneurysms due to the impact of high-pressure blood flow, which is further related to hemobilia. However, the specific causes and mechanisms still require more cases and studies to be obtained. It is hoped that this article can arouse the research interest of a wide range of scholars.

## Conclusion

IAPF, as a rare etiology of hemobilia, necessitates heightened clinical awareness to ensure timely diagnosis and appropriate intervention. Contrast enhanced CT and selective hepatic angiography remain pivotal for accurate identification. Endovascular embolization is the preferred therapeutic approach. Multidisciplinary collaboration is essential for optimizing outcomes. Further research is required to elucidate the underlying pathophysiological mechanisms.

## Data Availability

The original contributions presented in the study are included in the article/supplementary material, further inquiries can be directed to the corresponding authors.
